# The Serum Test for Typhoid Fever

**Published:** 1897-04-03

**Authors:** 


### THE SERUM TEST FOR TYPHOID FEVER.

The experience which is being accumulated in New York as to the utility, and also as to the limits of the utility, of the serum test for typhoid fever, is a matter of considerable interest, for there are few diseases in regard to which help in the diagnosis of doubtful cases would be more welcome. Although typhoid is often typical and plain as daylight, it is not infrequently so masked by its complications or imitated by other diseases as to render the diagnosis in early stages far from easy.

To what extent then will Widal's test help to clear the matter up? To make our answer clear a word must first be said as to the nature of the test. In the course of typhoid fever the blood acquires a property which it did not possess before, or, at least, only possessed in such small degree that it is practically a new attribute. This new property is that when it is added to a culture of living and active typhoid bacilli these microbes quickly cease their movements and become agglutinated in masses, leaving the fluid between them clear. There is no mistake about the reaction when it takes place. But what it is so important to understand is that the quickness and completeness with which it occurs depend largely upon the dose—upon the proportion existing between the culture and the blood.

It is therefore a quantitative test, and requires great judgment in its interpretation; for while, with but slight dilution, the reaction can be obtained in other diseases than typhoid, with great dilution even typhoid blood may show it in only very moderate degree. As the result of their experience in New York, Drs. Herman Biggs and William Park say *(American Journal of the Medical Sciences)* that it is possible, by the end of the first week of typhoid fever, to obtain by this means a positive diagnosis in fifty per cent. of the cases and a probable diagnosis in half the remainder, and they anticipate that the test will be found of value in many cases in which the diagnosis remains uncertain after the fifth day of the illness, and in the differential diagnosis of those irregular forms of the disease which are often difficult to distinguish clinically from it, such as typhus, malaria, miliary tuberculosis, malignant endocarditis, septicæmia, &c.

On the other hand it will not do to ignore the conclusions of Dr. V. Jez, whose investigations on the same subject (*Wien. Med. Wochensch.*, Jan. 16th, 1897) showed that in a case of tuberculous meningitis the agglomerating action of the serum was very distinct, and led him to think that this method of diagnosis could not be looked on as a perfectly reliable means of diagnosing typhoid fever.

But there is another aspect of the question which has to be borne in mind, for the agglutinating power, once set up, is apt to persist in the blood for a very considerable time after the disease has passed away; so that before expressing any positive opinion as to the meaning of the reaction, it is necessary to make sure that the patient has not had typhoid fever before. It is said that in some cases the power of producing this reaction still remained more than twenty years after having had typhoid fever.

While then we must accept Widal's test as a most useful help in doubtful cases, it will not do to admit that on the strength of it alone sanitary authorities should presume to over-ride the diagnosis of physicians who are conversant with the whole history and clinical details of the cases. As an aid in diagnosis it is good, as a substitute for it it is inadmissible.

